# The early history of cryo-cooling for macromolecular crystallography

**DOI:** 10.1107/S2052252519016993

**Published:** 2020-01-25

**Authors:** David J. Haas

**Affiliations:** a Tecco Corporation, 19 West Gate Road, Suffern, NY 10901, USA

**Keywords:** cryo-crystallography, cryo-protectant, flash-cooling, X-ray radiation damage, macromolecular crystallography

## Abstract

This paper recounts the first successful cryo-cooling of protein crystals that demonstrated the reduction in X-ray damage to macromolecular crystals. The project was suggested by David C. Phillips in 1965 at the Royal Institution of Great Britain and continued in 1967 at the Weizmann Institute of Science, where the first cryo-cooling experiments were performed on lysozyme crystals, and was completed in 1969 at Purdue University on lactate dehydrogenase crystals. A 1970 publication in *Acta Crystallographica* described the cryo-procedures, the use of cryo-protectants to prevent ice formation, the importance of fast, isotropic cryo-cooling and the collection of analytical data showing more than a tenfold decrease in radiation damage in cryo-cooled lactate dehydrogenase crystals. This was the first demonstration of any method that reduced radiation damage in protein crystals, which provided crystallographers with suitable means to employ synchrotron X-ray sources for protein-crystal analysis. Today, fifty years later, more than 90% of the crystal structures deposited in the Protein Data Bank have been cryo-cooled.

## Abstract 2

A description is given of the first protein crystals to be successfully cryo-cooled with cryo-protectants; the crystals were then exposed to intense X-rays with reduced damage. A history-of-science description is provided of cryo-procedures that are universally used today with synchrotron radiation for macromolecular crystallography.

## 1. Introduction  

to prolong the useful X-ray life of frozen … protein crystals

These words in the last paragraph of the April 1968 paper describing the first successful protein-crystal cryo-cooling experiments proved to be prophetic (Haas, 1968*b* ▸).

During the 1990s, the cryo-cooling of protein crystals (and other macromolecular crystals) became a universal technique for reducing the X-ray damage from synchrotron X-ray sources. This has been described remarkably well in many review papers by Garman, Pflugrath, Hope and others (Garman & Schneider, 1997 ▸; Garman, 2014 ▸, 2019 ▸; Gerstel *et al.*, 2015 ▸; Pflugrath, 2015 ▸; Hope, 1988 ▸). In April 1967, hen egg-white lysozyme crystals were successfully cryo-cooled in the Crystallography Department at the Weizmann Institute of Science, where I continued a project that began at the Royal Institution of Great Britain in 1965. Employing a simple cold gas stream (198 K) with a dry nitrogen coaxial stream from a single Dewar of liquid nitrogen, native lysozyme crystals with a sucrose cryo-protectant were exposed to the primary X-ray beam from a standard sealed Philips X-ray tube for several days without showing any noticeable radiation damage (as indicated by daily precession photographs). This was the first experiment to demonstrate that cryo-cooling could be successfully performed on protein crystals themselves, as well as reducing radiation damage to these crystals. A brief paper was published in 1968 in *Acta Crystallographica* describing these results (Haas, 1968*b* ▸).

**Figure 1:**
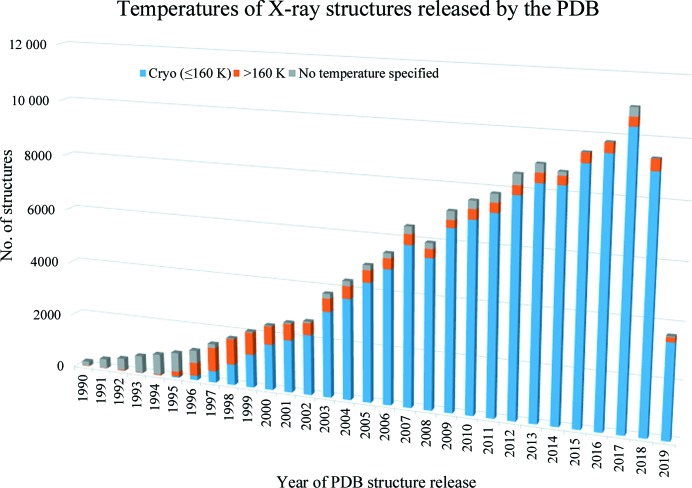
Histogram of X-ray crystal structures annually deposited in the PDB. The first structure determined from a cryo-cooled crystal (<160 K) was deposited in 1991 and such structures now represent almost 90% of deposited X-ray structures. (Unpublished data extracted from the PDB courtesy of Elspeth Garman and Markus Gerstel.)

Soon after the advent of the Six Day War on 5 June 1967, I left the Weizmann Institute for a new position with Michael Rossmann at Purdue University. The following year (1968) in the Rossmann laboratory, the cryo-cooling process (198 K) was again used with dogfish lactate dehydrogenase crystals (sucrose cryo-protectant). Employing new cryo-cooling apparatus identical to that used at the Weizmann Institute of Science, diffraction intensities were collected to 3.5 Å resolution on a Picker four-circle diffractometer and the data were successfully processed. Two reference reflections were monitored throughout the three months of experimental work, which demonstrated that compared with equivalent room-temperature crystals, cryo-cooled crystals reduced the radiation damage more than tenfold. After the data collection was complete and processed, we wrote a paper describing this cryo-cooling work (Haas & Rossmann, 1970 ▸). Basically, it demonstrated that cryo-cooling may be a means for collecting complete diffraction data from a single protein crystal. [This had been the goal of the original project suggested by David C. Phillips in 1965 at the Royal Institution of Great Britain.] It required more than 20 years for cryo-cooling procedures to become routine, with Garman and others spending years developing and perfecting cryo-cooling techniques, to the extent that most of today’s macromolecular diffraction data are collected at 100 K. This has resulted in more than 90% of all macromolecular crystal structures deposited in the Protein Data Bank now being derived from cryo-cooled samples (Fig. 1 ▸).

This ‘history-of-science’ paper will describe the means and the sequence of the experiments that were performed. It also explains the background as to why the project was originally proposed by David Phillips in 1965.

## 2. The beginning of the radiation-damage reduction project   

After my graduate work with David Harker (Tulinsky, 1996 ▸) and graduating from the University of Buffalo in February 1965 (Haas, 1965 ▸), my wife and I traveled to London in September 1965 for postdoctoral work at the Royal Institution of Great Britain. David Phillips and the lysozyme group (Gareth Mair, Colin Blake, Louise Johnson, Tony North and Ragupathy Sarma) were just finishing the structure of lysozyme, the first protein structure of an enzyme (David Phillips was then writing the article for *Scientific American*). At that time, Sir Lawrence Bragg was the Director of the Royal Institution. Within the first few days, I met with David Phillips to discuss suitable research projects. He suggested a project for reducing the radiation damage to lysozyme (and other) protein crystals. Lysozyme crystals could be cross-linked with glutaraldehyde to make them more robust (Quiocho & Richards, 1964 ▸), and this may reduce the X-ray damage. This bireactant aldehyde connects nearby lysine side chains on the protein surface (intermolecularly and intramolecularly) and forms a single crystal consisting of millions of lysozyme molecules covalently bound together. He hoped that a successful result could produce a universal means to reduce X-radiation damage in protein crystals so that a complete set of diffraction data could be collected *from a single crystal*. No other means of reducing radiation damage had ever been demonstrated. Hence, this challenging project could be very useful, as protein crystallo­graphers were currently required to replace their experimental crystals at room temperature frequently after only a few hours of data collection. David Phillips had first-hand experience with this issue from his 1962 work that had clearly demonstrated radiation damage in myoglobin crystals (Blake & Phillips, 1962 ▸). The radiation damage to protein crystals is continuous from the first exposure to the X-ray beam, and as the protein-crystal damage increases, the intensity of the diffraction beams from these damaged crystals slowly weakens, with the higher resolution reflections decreasing faster. This non-uniform change in the diffracted beams presents a major problem for scaling the data between several crystals (Arndt & Phillips, 1961 ▸). David Phillips was the first to use a real-time automated diffracto­meter for protein crystal data collection and he experienced this problem while using the group’s linear diffractometer, the first automated diffractomer.

**Figure 2:**
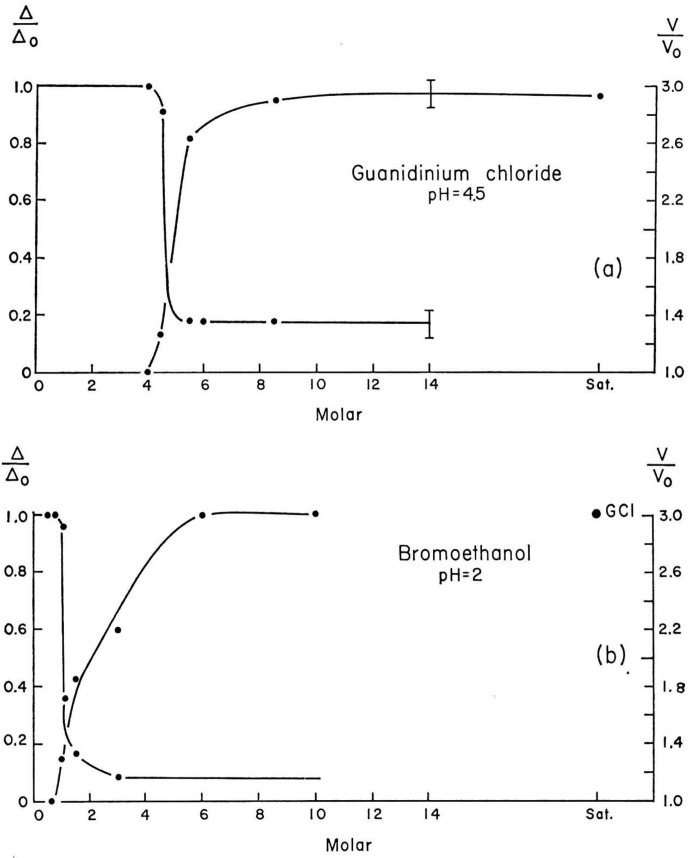
Volume increase of ‘crystal to gel’ upon the denaturation of cross-linked lysozyme crystals. The graph shows normalized optical retardation and crystal volume for fully cross-linked lysozyme crystals with increasing concentrations of denaturants (reproduced from Haas, 1968*a* ▸, with permission from Elsevier).

**Figure 3:**
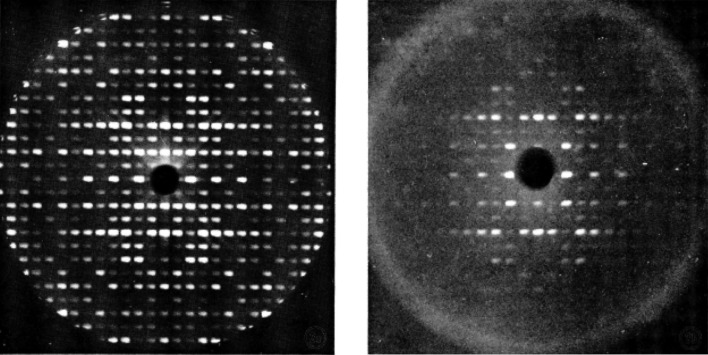
Precession photographs of a native lysozyme crystal and a ‘renatured’ cross-linked lysozyme crystal. The left-hand photograph is a 9° precession photograph of native lysozyme. The right-hand photograph is after the crystal was ‘swelled’ by denaturation and renatured in the original supernatant (reproduced from Haas, 1968*a* ▸, with permission from Elsevier).

With an ample supply of surplus lysozyme crystals from the lysozyme group, I first tested cross-linking the crystals using different concentrations of glutaraldehyde. Lysozyme was an excellent test model as the crystals were very stable and rugged (Haas, 1967*a* ▸). Most of the lysozyme crystals I used throughout my work had nice shapes with sharp edges and were between 0.5 and 1 mm in size: easy to view in a light microscope or even by eye. A measure of the ‘degree of cross-linking’ was to denature the lysozyme crystal and observe the volume of swelling. The lower the cross-linking level, the greater the swelling. For example, brief exposure to a dilute solution of glutaraldehyde produces only ‘surface cross-linking’. These crystals, when denatured, swelled to enormous sizes, with each crystal edge increasing more than three times (Fig. 2 ▸; Haas, 1967*b* ▸, 1968*a* ▸). It is basically a swelled covalent gel. I compared these surface cross-linked crystals with fully cross-linked crystals and found that the ‘surface’ cross-linked crystals appeared to be unchanged internally and basically acted like native crystals except that they did not dissolve because of the cross-linked ‘skin’ on the crystal. Most importantly, with denatured cross-linked lysozyme crystals (swelled, both fully and surface cross-linked), slowly removing the denaturant and returning the crystal back to its original supernatant caused the ‘gel’ crystal to shrink again and recover its X-ray diffraction pattern (Fig. 3 ▸). This proved to be a remarkable renaturation property which shows that the protein molecules can actually rearrange/recrystallize themselves. I presented several papers on this remarkable property at European crystallographic meetings and published a short note in the *Biophysical Journal* (Haas, 1966 ▸, 1968*a* ▸).

The Lysozyme Radiation Damage Project consisted of using only surface cross-linked lysozyme crystals, adding different solutes to the mother liquor (over 24 h) with only a single crystal in the solution and then placing the lysozyme crystal in an X-ray beam for two days (typically 60 kV at 40 mA with a Philips standard generator: a four-port beryllium-window copper-anode X-ray tube). Typically, the solute concentration was 30% in order to ensure that sufficient solute was present to have an effect (the solutes were all nonreacting and consisted of many inorganics, salts, nondenaturing organics, sugars *etc.*). With the two-day X-ray exposure, significant intensity changes were always visible, and I made a reference diagram of the *hkl* spots to evaluate each precession photograph. The first experiments performed were radiation-damage tests on fully crossed-linked lysozyme crystals. These crystals showed the same decay of radiation-damage diffraction patterns as native crystals, so the conclusion was that *cross-linking alone did not provide any radiation protection.*

With each of the surface cross-linked crystals in a different solute solution, each crystal was mounted in a sealed Lindemann glass tube, an initial precession photograph was taken and a second photograph was taken typically two days later. Every photograph was taken with exactly the same settings and these precession photographs showed the usual 5–10 diffraction spots that changed in a characteristic pattern. Hence, improved radiation protection (less damage) should be easily recognized. During the 15 months of work at the Royal Institution, dozens and dozens of different solutes were tested, but there was never a single one that demonstrated reduced radiation damage. I worked alone on this project for the entire time at the Royal Institution.

## 3. Cryo-cooled lysozyme crystals at the Weizmann Institute of Science   

In the fall of 1966, David Philips informed me that he was moving to Oxford University and that I would need to find another laboratory to continue my NIH Fellowship. He suggested the Weizmann Institute of Science in Israel, and said he would contact Wolfie Traub, who was the head of the Crystallography Group in the Chemistry Department. He knew both Wolfie Traub and Gerhard Schmidt from scientific meetings and prior visits to the Weizmann Institute.

This was a satisfactory arrangement for me, so my wife and I traveled to Israel in December 1966. As my NIH Fellowship was not transferable, the Weizmann Institute provided me with a Weizmann Fellowship which included both housing and a stipend (what luck!).

Being motivated and still considering the project worthwhile, I took all of the lysozyme crystals and supplies with me so I would be ready to continue the work at the Weizmann Institute. It was re-established in the crystallography laboratory, where they had all of the necessary equipment. The work continued for another three months, but finally, sometime in March 1967, I reviewed all of the results and concluded that neither cross-linking nor any of the many dissolved solutes provided any radiation protection for these lysozyme crystals. Surely with all of the different solutes tested over the past 18 months, at least one of them should have shown some reduction in radiation damage. This was quite disappointing!

To redirect the radiation-damage project, I prepared a list of all the alternative means that I could think of and reviewed the list with Wolfie several times (Table 1 ▸). One item on this list of alternatives was freezing (now called cryo-cooling), which offered two special benefits: firstly, since radiation damage is the result of electrons being ejected from the atoms of the molecules in the crystal, cryo-cooling may immobilize all of these ejected electrons and possibly prevent the free radicals from moving around; secondly, even if intramolecular damage did occur, the frozen (immobile) state of the protein molecules and the surrounding vitrified water would keep the structures in place and immobilize the entire crystal. Hence, all of the atoms in the immobilized protein molecules could remain as coherent scattering centers. This seemed to be reasonable.

**Figure 4:**
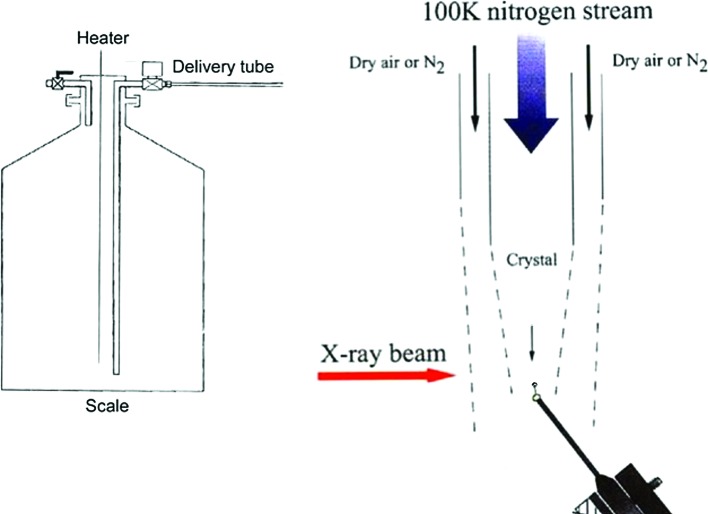
Cryo-cooling apparatus design used at the Weizmann Institute of Science in 1967 (Haas, 1968*b* ▸; modified from Garman & Schneider, 1997 ▸).

As fate would have it, there was an unused crystal cryo-cooling apparatus on one of the Philips X-ray generators in the laboratory which Wolfie had constructed several years earlier (Post *et al.*, 1951 ▸; Rudman, 1976 ▸; Traub & Hirshfeld, 1960 ▸). The cryo-apparatus consisted of a double glass tube to deliver cold nitrogen gas around the crystal with an outer tube for room-temperature nitrogen gas (Fig. 4 ▸). The single Dewar tank had a heater in the bottom and a single delivery pipe for the cold gas. I set the correct milliamperage to the heater to provide a constant gas flow that I used for all experiments. Each afternoon, I would refill the Dewar for overnight operation. There was little ice formation, which if present would have been a major problem, but I now attribute this to the low humidity in that part of Israel (more luck!). This apparatus provided me with an ideal opportunity which I probably would not have had at any other laboratory. More than this, the current laboratory technician was the individual who actually constructed the cryo-cooling apparatus, and Wolfie suggested that I let him teach me how to use it. (Again, what luck! Now I believe Wolfie was quite pleased to have his cryo-apparatus being used again.)

A flow chart on how to proceed was made while setting up the crystal cryo-cooling apparatus.

Firstly, of course, just cryo-cool a native lysozyme crystal in its aqueous supernatant as a baseline to show that ice would surely form and destroy the protein crystal. At the time, I was unaware of any previous cryo-cooling papers on protein crystals (King, 1958 ▸; Low *et al.*, 1966 ▸), and now I wonder whether knowledge of these papers would have discouraged me. Obviously, I knew from the beginning that some type of antifreeze (now called cryo-protectant) would be needed, so the natural choice was glycerol, which is completely water-soluble and was known not to be a denaturant (from my previous experiments). During the months of April and May 1967, the seven different experiments described below were performed.

### 3.1. Experiment 1: observe the effect of cryo-cooling (198 K) and X-ray exposure on cryo-cooled lysozyme crystals (cross-linked) in aqueous supernatant   

Once I had the cooling apparatus operating and stable for hours at a time, I mounted the first lysozyme crystal in the glass capillary directly from the supernatant without any cryo-protectant. Even though the lysozyme crystal was surface cross-linked, I knew it would freeze with ice crystals, but I had never seen this before. With the cold nitrogen gas enveloping the centered position on the precession camera and producing a steady cryo-stream (198 K) for about half an hour, I inserted the goniometer head into the precession camera (I would always center the crystals on the precession camera spindle before turning on the liquid-nitrogen gas; this ensured that the crystal inside the capillary would be exactly in the gas jet and would cryo-cool instantly). Because of the fluctuations of the nitrogen gas steam while refilling the feed Dewar, a general practice was to stop the exposure during liquid-nitrogen refills. This proved to be a wise practice, and I followed the same procedure a year later during the Purdue University work. Also, the Dewar was on a bathroom scale, so I knew if it had to be refilled that night.

In the cold nitrogen stream, the lysozyme crystal turned from being clear to translucent, so I knew that ice had formed in the crystal. After developing the precession photograph, the ice-crystal pattern and circular ice powder diffraction lines were obvious. There were only a few diffraction spots from the lysozyme crystal itself. It was evident that the crystal needed a cryo-protectant. Hence, I already had soaked several cross-linked lysozyme crystals in 30% glycerol so that I was ready for the next experiment.

### 3.2. Experiment 2: observe the effect of cryo-cooling (198 K) lysozyme crystals with cryo-protectant (cross-linked, 30% glycerol)   

Now that an X-ray exposure routine with the cryo-apparatus had been established, I mounted a lysozyme crystal in a capillary (cross-linked, 30% glycerol), centered it on the precession camera for the correct position in the nitrogen gas jet and then removed it. The cryo nitrogen gas jet was then turned on and after about 30 min I inserted this aligned goniometer head onto the precession camera. The lysozyme crystal cooled and remained clear. The usual precession photograph exposure time for these crystals was about 30 min.

There was no way of knowing what to expect with this first precession photograph. Would it show only ice, would it show split diffraction spots from the lysozyme crystal fracturing, or maybe something else? So, with the developed precession photograph in hand, I looked at it just as I had looked at dozens of previous lysozyme photographs. This first cryo-cooled lysozyme precession photograph was pristine, exactly like normal room-temperature lysozyme precession photographs. At first, it may have been what was expected because (as always) it was just another perfect lysozyme precession photograph. Then I realized that there was no ice pattern, and the lysozyme pattern was surprisingly normal. I remember asking myself if this was what I expected? I had not thought that far ahead as I expected the usual failure.

### 3.3. Experiment 3: observe the effect of several days of X-ray exposure on cryo-cooled (198K) lysozyme crystals (cross-linked, 30% glycerol)   

The plan was to leave this first lysozyme crystal in the X-ray beam for several more days, just as I had been doing with the dozens of previous room-temperature samples. As every radiation-damage experiment performed in the past 18 months had shown radiation damage (changes in the diffraction-pattern spots), I recall being so surprised that cryo-cooling this crystal had not been a failure. Well, I would just have to wait several days before getting excited about a successful experiment, having actually found a means that might work.

The next day, being the patient individual that I am, I took another precession photograph *just to be sure that nothing had changed*. This photograph after 24 h of X-ray exposure looked just as perfect as the first day’s. And so, I waited another long day and repeated the precession photograph again. It too showed none of the usual intensity changes that were always present after two days of X-ray exposure.

**Figure 5:**
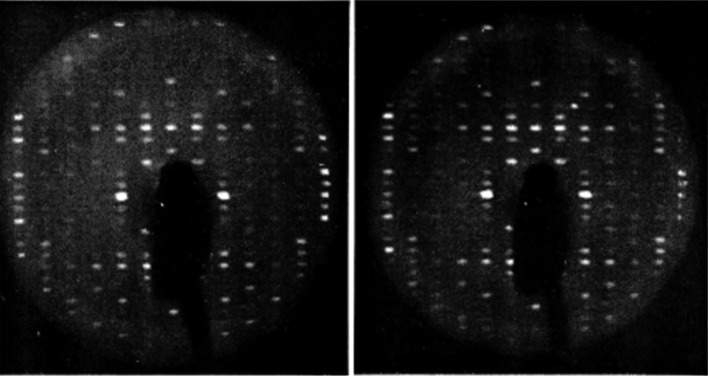
The left photograph is a 9° precession photograph of a native lysozyme crystal with sucrose cryo-protectant at about −50 C. The right photograph is the same cryo-cooled crystal after several days of X-ray exposure (reproduced from Haas, 1968*b* ▸).

It was hard to believe that just cryo-cooling alone would provide such good radiation protection. Since I had made no plans on what to do after this, I decided that the next experiment should be with native crystals without cross-linking, since cross-linking was such a special treatment that probably would not work well with other protein crystals. I recalled that David Phillips had said that the ultimate goal of the project was to ‘routinely collect’ an entire set of diffraction data from a single crystal. So, it was important to make this process simple and routine. In the meantime, this first lysozyme crystal was just left in the X-ray beam while spending several days preparing for the next experiment. It was days later that a final precession photograph was taken from this first cryo-cooled lysozyme crystal – with still no apparent radiation damage (Fig. 5 ▸)! Success?

### 3.4. Experiment 4: observe the effect of cryo-cooling (198 K) ‘native’ (non-cross-linked) lysozyme crystals in 10/20/30% glycerol   

A number of solutions with varying amounts of glycerol were prepared, at concentrations such as 10, 20 and 30%(*v*/*v*). Since these were going to be native (non-cross-linked) lysozyme crystals, the solutions had to be changed slowly. Over the next few days this was performed, but in the end all of the native crystals dissolved in the glycerol solutions. Some of the papers I had read on antifreeze solutions and antifreeze alternatives to glycerol discussed sugars, and since sucrose (and several other sugars) were used in my previous radiation-damage work at the Royal Institution, I knew that sucrose was a very safe choice; it had never shown any deleterious effect on lysozyme crystals.

### 3.5. Experiment 5: observe the effect of cryo-cooling (198 K) ‘native’ (non-cross-linked) lysozyme crystals in 30% sucrose   

Once again, the protein solutions were slowly changed to bring the supernatant to 30%(*v*/*v*) sucrose at room temperature. (My previous experiments with sucrose solutions at room temperature had shown no radiation-damage protection.) With these native crystals soaked and mounted in glass capillaries, they were cryo-cooled (198 K) in the cold nitrogen stream on the precession camera and a first precession photograph was taken. The cryo-cooled crystal remained clear, presumably indicating no ice formation. The first developed precession photograph presented a perfect diffraction pattern with no visible ice pattern. X-ray exposure was continued for several more days.

### 3.6. Experiment 6: observe the effect of several days of X-ray exposure on cryo-cooled (198 K) ‘native’ (non-cross-linked) lysozyme crystals in 30% sucrose   

After two days of continuous X-ray exposure, once again the diffraction pattern showed no apparent differences from the first photograph taken at the time of cryo-cooling. This convinced me that yes, apparently radiation damage can be reduced or stopped by simply cryo-cooling the protein crystals. It was early May 1967; I had sent my wife and son back to our friends in England owing to the war hysteria. I was the only person in the laboratory (in the entire building), and I finally realized that this was a eureka moment! But there was no one to tell.

Since this particular crystal showed no radiation damage after two days and gave excellent precession photograph patterns, I decided just to let the X-ray exposure continue for the remainder of the week. (There were only a few other people in the entire Institute, so what else did I have to do?) In the meantime, I wanted to reduce the cryo-protectant (sucrose) to as low as possible, so I prepared more lysozyme crystals with less sucrose, probably in the 5–10%(*w*/*v*) range. This would convince me that as long as there was NO ice formation, cryo-cooling would be a useful solution to the radiation-damage problem for protein crystals. So, after another week, the sucrose-protected lysozyme crystal still showed no change in the precession photographs, and I knew that this phenomenon was real! Eureka!

### 3.7. Experiment 7: observe the effect of several days of X-ray exposure on cryo-cooled (198 K) ‘native’ (non-cross-linked) lysozyme crystals in minimal sucrose as the cryo-protectant   

These final experiments were performed during the last days of May 1967. Since almost all of the staff and employees of the Weizmann Institute were not available, I worked alone in the laboratory. I also had to transport the liquid-nitrogen Dewars back and forth between the maintenance-department supply tank and the crystallography laboratory.

The next plan was to obtain crystals of other proteins so that I could demonstrate that this cryo-cooling process might be universally applicable, but there was no one available to provide samples. So, I tested a few other parameters, such as soaking crystals in organic solvents (Haas, 1969 ▸) and warming some of the cryo-cooled crystals to room temperature and demonstrating that they remained as a single crystal, producing good diffraction patterns. All of these cryo-cooled and warmed lysozyme crystals continued to produce good diffraction patterns. Apparently, turning the liquid water in protein crystals into a vitreous solid and then back into a liquid was not ‘seriously’ detrimental to lysozyme crystals and did not produce a disruptive volume increase.

My work ended on 5 June 1967 and conversations with several Weizmann Institute employees indicated that serious work at the Institute would not resume for several months. Fortunately, there were already sufficient precession photographs for publication, demonstrating that cryo-cooling substantially reduces radiation damage. Since arrangements for a position with Michael Rossmann at Purdue University had already been established for whenever I returned to the United States, I contacted Michael to confirm this. Hence, I decided to return to the United States in June. This proved to be a good decision.

If not for these fortuitous events, the proof of ‘reduced radiation damage’ by cryo-cooling crystals would probably have been delayed for years. Also, my follow-on work with Michael Rossmann would definitely not have happened as Michael was only convinced by these lysozyme precession photographs from the Weizmann Institute. I was sure that work at Purdue would provide quantitative evidence (proof) for reduced radiation damage.

## 4. Proof of reduced radiation damage at Purdue University   

The Rossmann laboratory was a beehive of activity, with Margaret Adams, Alan Wonacott, Michael Schevits and Alex McPherson all working on the lactate dehydrogenase (LDH) project (Wonacott *et al.*, 1968 ▸). Michael had the latest equipment and the group was always willing to help me learn the new methods. A short paper describing my Weizmann Institute findings for reduced radiation damage was written on the return trip to the United States, so this was given to Michael and the other members of the Rossmann group to support my project proposal (Haas, 1968*b* ▸).

Everyone was skeptical of the benefits of extending the useful life of the crystals: would it be worth the ‘perceived’ complexity and difficulty of building, installing and operating cryo-apparatus? They assumed that the radiation-damage reduction would be only a few hours, not the hundreds of hours that finally came to be shown. (And certainly, no one could even imagine the importance of cryo-cooling crystals with synchrotron-radiation sources.) Again, the only convincing demonstration of reduced radiation damage were the lysozyme precession photographs from the Weizmann Institute. The general notion at the time was that cooling protein crystals was no different from freezing food. They believed that freezing anything, in general, was a bad idea!

After weeks of discussion, Michael agreed to loan me one of the Picker automated diffractometers for three months (and no more!) in the spring of 1968, and he would fund the purchase and construction of the most primitive cryo-cooling apparatus if I put the system together myself. (It probably would only be used once for my project anyway.) The Purdue glass workshop fabricated a co-axial gas-delivery jet for directing the nitrogen gas onto the crystal with a dry air outer barrier jet surrounding the nitrogen stream. I made it exactly like the Weizmann Institute apparatus. Initially, the single crystals were each mounted in a Lindemann glass vial in the conventional manner along with a small amount of mother liquor, but later on I simply cooled the crystals positioned near the end of a single glass fiber. This was a major step forward (I never thought of adding a loop at the end; Teng 1990 ▸). Michael wanted the work to be performed on lactate dehydrogenase and sufficient crystals were available (this proved to be an important decision!). Sucrose was tested as a cryo-protectant for lactate dehydrogenase as soon as the cryo-equipment was ready: it worked perfectly.

**Figure 6:**
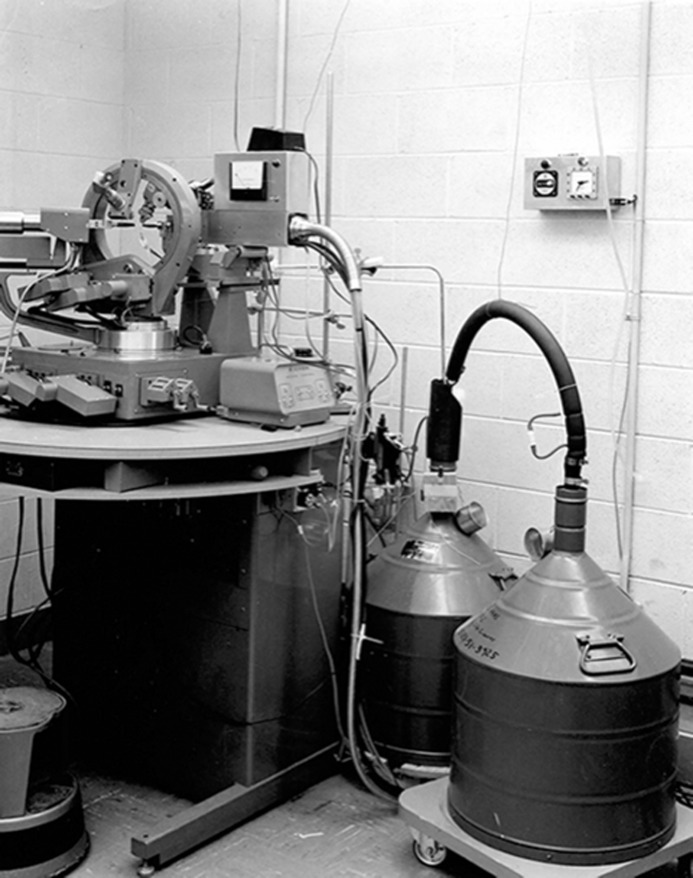
The cryo-cooling apparatus used at Purdue University in 1968 for lactate dehydrogenase data collection (Haas & Rossmann, 1970 ▸). The Dewar on the left is as shown in Fig. 4 ▸, while the Dewar on the right is transport for refilling. (Photograph by D. Haas.)

A temporary setup was rigged up on a laboratory precession camera and this was successful for obtaining precession photographs of cryo-cooled LDH crystals. So, after Michael approved the project in late 1967, the cryo-apparatus was ready to perform the data collection in spring 1968 (Fig. 6 ▸). The Purdue Laboratory Supply group was very helpful. With the cryo-gas tube assembly ready, simple control circuits were fabricated and we purchased a suitable air dryer and several bathroom scales for tracking the weight of the liquid-nitrogen-filled Dewars (the scale weight always showed the remaining liquid nitrogen in the Dewar). Three months of work were required for the data-collection portion of the project and data collection began in April 1968.

Quoting from the 1970 paper (Haas & Rossmann, 1970 ▸)

Cooling in a jet of cold gas was equally unsuitable because of more rapid cooling of the crystal faces near the gas jet. Thus, the usual mounting procedure consisted of placing a single LDH crystal (approximately 0.5 mm of each edge) on a strip of filter paper with a dropper, waiting until the liquid had been drawn off by the paper, scooping the crystal up on the end of a 0.25 mm glass fiber, and immediately plunging it into, and retaining it in liquid nitrogen. The crystal was now frozen to the fiber and completely immobile. As the fiber had previously been mounted and aligned on the goniometer head, the crystal was also nearly centered for the diffractometer. Finally, it was transferred quickly from the Dewar full of liquid nitrogen to the diffractometer where a jet of cold nitrogen gas prevented thaw. Ice formation was prevented both by use of a co-axial room-temperature nitrogen jet and by surrounding the whole diffractometer with a dry-nitrogen filled polyethylene bag.

Regarding the estimated cryo-temperature of the LDH crystals, a statement in the Haas and Rossman paper says

A thermocouple placed near the end of the cold jet provided a continuous record of the crystal temperature.

**Figure 7:**
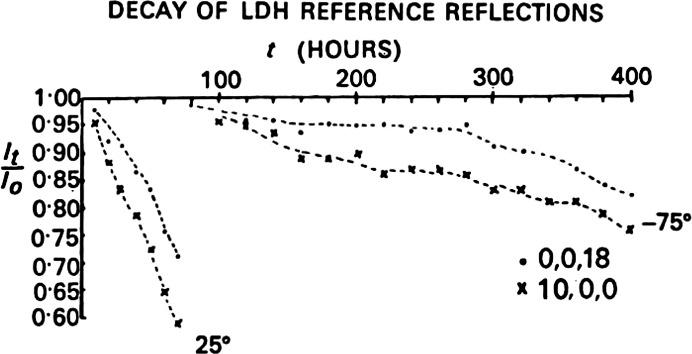
Original graph showing two reference reflections from lactate dehydrogenase crystals at room temperature and cryo-cooled. The cryo-cooled crystals show more than a tenfold reduction in radiation damage (reproduced from Haas & Rossmann, 1970 ▸).

I believe that the stated cryo-temperature of −75 C was only an estimate; I do not remember how a calibration was performed. The data collection was continuous from the first day, with only a few interruptions when the mechanical encoders of the Picker diffractomer broke. We also collected data from LDH crystals at room temperature in order to prepare the radiation-damage reference reflection graph (Fig. 7 ▸).

The Picker diffractometer was controlled by punched cards and it was run 24 hours a day. The Picker ‘mechanical’ shaft encoders were most unreliable and had to be repaired and replaced several times. Otherwise, I fell into a routine for several months of loading the punched cards, filling the Dewar and regularly checking the two reference reflections to ensure that the crystal was properly aligned.

We processed the data during the summer and fall of 1968 and Michael spent several months analyzing the electron-density maps (Adams *et al.*, 1968 ▸; Haas & Rossmann, 1968 ▸). The data produced nice results, with the most important graph showing the reduced intensity decay of the two reference reflections (Fig. 7 ▸). This radiation-decay graph indicated that the radiation damage in cryo-cooled LDH crystals was reduced more than tenfold at 198 K compared with room temperature. It was the first quantitative data that we had for radiation-damage reduction. Michael wrote the final draft of the paper and submitted it to *Acta Crystallographica* in July 1969. (The tenfold reduction was low, probably because of the varied cooling temperature of the crystals.) Reduced radiation damage on cryo-cooling crystals was independently confirmed by Gregory Petsko in his 1975 study on protein crystals at subzero temperatures (Petsko, 1975 ▸).

During 1969, after interviewing for positions at several universities and pharmaceutical companies, a perfect industrial position became available. I joined the electronics industry as an X-ray scientist (Philips Electronic Instruments in Mount Vernon, New York), ending my protein crystallo­graphy career and, within two years, ending my work in X-ray crystallography altogether.

## 5. Cryo-crystallography and the AIDS Lazarus effect   

On being invited to give the weekly Rutgers Protein Data Bank Seminar on 24 April 2019, I reviewed the extensive literature regarding the history and chronology of macromolecular cryo-crystallography (Garman & Schneider, 1997 ▸; Garman & Weik, 2015 ▸; Hope, 1990 ▸; King, 1958 ▸; Low *et al.*, 1966 ▸; Petsko, 1975 ▸; Pflugrath, 2015 ▸). There were many papers on drug design and on the development of structure-based drug design in particular. As I stated earlier, cryo-cooling was slow to be implemented, even though a number of crystallographers experimented in the field between 1975 and 1990. After 1970, it appears that crystallographers generally knew the benefits of cryo-cooling. The development of structure-based drug design was reviewed frequently (Goodford, 1984 ▸) and developed as early as 1978, even before the 3D protein structures of target molecules were available. Structure-based drug design has been a motivating force for the pharmaceutical industry, and with the AIDS epidemic in the 1980s, protein crystallography, synchrotrons and cryo-cooling had already become a major force for attacking this issue (Wright, 2009 ▸).

**Figure 8:**
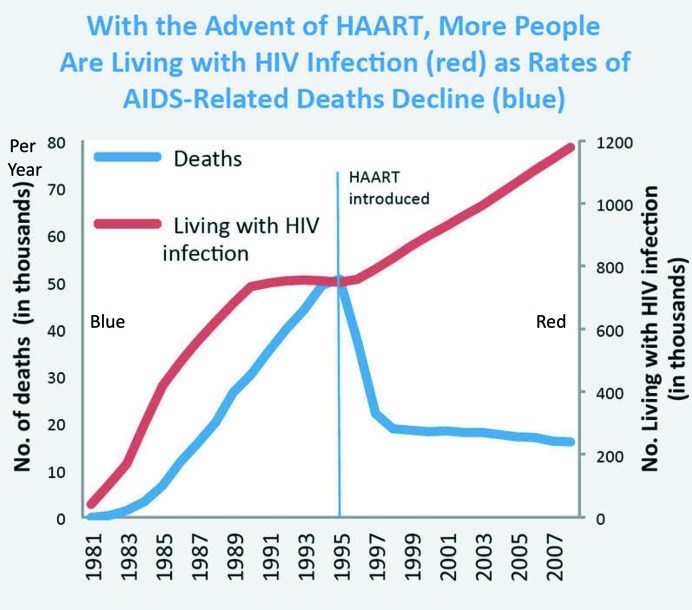
Graph showing the Lazarus effect in 1996 with the growth of the AIDS epidemic in the United States (1980–1996) and the introduction of HAART (highly active antiretroviral therapy, now renamed ART) in 1996 (CDC Mortality in the United States).

In early 2017, I was fortunate to be invited to attend the Cold Spring Harbor X-Ray Methods Course. In addition to this being my first direct exposure to modern crystallography, I was able to participate in laboratory work employing cryo-crystallography. However, most significantly during this intense 16-day experience, one of the instructors told me ‘the early invention of macromolecular cryo-crystallography certainly advanced the field by 10–20 years’. I never thought much about this until I began reading about the ‘AIDS Lazarus Effect’ (Fig. 8 ▸; Cold Spring Harbor Laboratory, 2016 ▸). The AIDS Lazarus effect is just one example of the success of cryo-crystallography, which has contributed substantially to structural biology.

**Figure 9:**
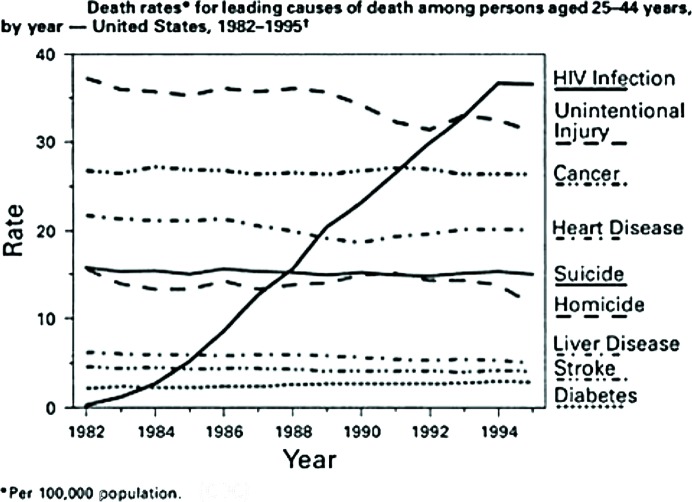
Graph showing the leading causes of death in the United States (for the years 1980–1996) and the epidemic curve for HIV infections (CDC Mortality in the United States).

The medical profession knows the Lazarus effect as a disease situation in which the mortality rate is very high, but suddenly an event occurs that enables vast numbers of sick people to live. The AIDS epidemic began in about 1980 and is still progressing today (see the World Health Organization AIDS Reports). However, between 1980 and 1996, individuals in the United States who became infected with HIV had only a 4% survival rate (Fig. 9 ▸). With 96% of HIV-infected individuals dying within 2–4 years, the United States was the first country to experience thousands of AIDS deaths, and the total deaths projected during the next few decades was expected to be several million.

The intense work of the NIH, independent laboratories and pharmaceutical companies produced a remarkable therapy (not a cure). By 1989, the 3D crystallographic structure of HIV1 protease had been determined and the structure was made available to the public for all scientists to use (by Merck Pharmaceutical, New Jersey; Navia *et al.*, 1989 ▸). Beginning in about 1985, structure-based drug design had become a serious process for drug discovery, and using synchrotrons, superior computers/software and cryo-crystallography, numerous pharmaceutical companies targeted HIV protease for the development of drug inhibitors. Not a single drug before 1995 had reduced the death rate or proved successful against the AIDS epidemic. The worldwide infection rate, including in the United States, had continued to grow.

The first HIV inhibitor, saquinavir, was approved by the FDA in 1995 and three more inhibitors were approved by the FDA within months (Harden, 2012 ▸). This first HIV treatment was called HAART (highly active anti-retroviral therapy, later renamed ART for anti-retroviral therapy) and was a combination of three different drugs, with successful testing beginning in about 1994. The HIV protease inhibitor was the third component of the cocktail that made it work! Clinical trials proved successful for the HAART treatment and, after FDA approval, HAART was introduced in quantity in 1996. The HIV/AIDS death rate plummeted from 96% to below 20% within a few years. (Imagine being one of tens of thousands HIV-infected individuals going to your doctor and being told that your HIV infection is no longer a death sentence, but a manageable disease.) The HIV Lazarus effect is shown in Fig. 8 ▸ as the steep decline in the death-rate curve (blue).

[To explain Fig. 8 ▸, which shows AIDS-related deaths between 1980 and 1996, the individuals on the steep red line (HIV-infected) died within 2–4 years and their count moved onto the blue line (deaths). In 1996, thousands of individuals on the red line (HIV infections) did not die and hence simply increased the slope of the HIV-infected curve (red line). This produced a dramatic decrease in deaths (the steep decrease in the blue line after 1996) and hence a substantially reduced death rate. These HIV-infected individuals have now lived for decades with this manageable disease.]

It is estimated that from 1996 to date, 862 000 Americans have been saved from a premature death by this drug therapy (PhRMA, 2018 ▸). Using the official Centers for Disease Control and Prevention (CDC) data, it appears that this early cryo-cooling crystallography procedure for structure-based drug design may have saved the lives of 250 000 Americans. This is remarkable. (The worldwide AIDS epidemic is still ongoing and, contrary to the decline in death rates for Americans, the rate worldwide has not decreased significantly; see the World Health Organization AIDS Reports).

For me, this story is a real back-to-the-future experience. David Phillips would be (and should be) very pleased! Furthermore, most science is not time-sensitive, but the 750 000 HIV-infected Americans in 1996 did not have the luxury of waiting; the ten years advance from cryo-crystallo­graphy saved them. They were very fortunate. This is truly ‘Science for the Benefit of Humanity’ (the Weizmann Institute of Science phrase).

I want to thank, once again, the instructors of the Cold Spring Harbor Laboratory X-ray Methods Course and specifically Elspeth Garman, Alex McPherson, James Pflugrath and Stephen Burley of the Rutgers Protein Data Bank for inviting me to tell this story, and of course my wife Sandra. Discovering what actually happened fifty years ago has certainly been an exciting STEM experience. And there were many fortuitous circumstances (luck) that ‘just made it happen’!

## 6. Links   

Cold Spring Harbor Laboratory *LabDish* Blog article, 24 January 2018: https://www.cshl.edu/45-years-later-scientist-realizes-hes-star/.

ACA Living History biography by the American Crystallographic Association, January 2016: http://www.amercrystalassn.org/h-david-haas.

## Figures and Tables

**Table 1:** Alternate means to reduce radiation damage in protein crystals (April 1967)

1. Add free-radical scavengers
2. *Freeze crystals to immobilize agents (causing radiation damage)*
3. Increase the rigidity of protein crystals (cross-linkers)
4. Replace water (organic liquids)
5. Use cavity fillers (polymers)
6. Use some kind of glue (between protein molecules)
7. Water replacement (polyhydroxy liquid)
8. Other (?)

## References

[bb1] Adams, M. J., Haas, D. J., Rossmann, M. G. & Wonacott, A. J. (1968). *Experience on Low Temperature Data Collection for Lactic Dehydrogenase at −75°C*. Abstract A3. American Crystallographic Association.

[bb2] Arndt, U. W. & Phillips, D. C. (1961). *Acta Cryst.* **14**, 807–818.

[bb3] Blake, C. C. F. & Phillips, D. C. (1962). *Biological Effects of Ionizing Radiation at the Molecular Level*. Vienna: International Atomic Energy Agency.

[bb4] Cold Spring Harbor Laboratory (2016). Meeting on HIV/AIDS Research: Its History and Future, 13–16 October 2016. Cold Spring Harbor, New York, USA.

[bb5] Garman, E. F. (2014). *Science*, **343**, 1102–1108. 10.1126/science.1247829 24604194

[bb6] Garman, E. F. (2019). *Biophys. Rev.* **11**, 539–541. 10.1007/s12551-019-00566-7 PMC6682181 31278590

[bb7] Garman, E. F. & Schneider, T. R. (1997). *J. Appl. Cryst.* **30**, 211–237.

[bb8] Garman, E. F. & Weik, M. (2015). *J. Synchrotron Rad.* **22**, 195–200. 10.1107/S160057751500380X 25723921

[bb26] Gerstel, M., Deane, C. M. & Garman, E. F. (2015). *J. Synchrotron Rad.* **22**, 201–212. 10.1107/S1600577515002131 PMC4344357 25723922

[bb9] Goodford, P. J. (1984). *J. Med. Chem.* **27**, 557–564. 10.1021/jm00371a001 6716396

[bb10] Haas, D. J. (1965). *Crystal Structures of Complexes Between Protein Denaturants and Model Peptides*. Thesis. State University of New York at Buffalo, Buffalo, USA.

[bb12] Haas, D. J. (1966). *Studies and Denaturation of Cross-linked Lysozyme Crystals*. International Union of Pure and Applied Physics, Vienna.

[bb13] Haas, D. J. (1967*a*). *Acta Cryst.* **23**, 666.

[bb14] Haas, D. J. (1967*b*). *Isr. J. Chem.* **5**, IVp.

[bb15] Haas, D. J. (1968*a*). *Biophys. J.* **8**, 549–555. 10.1016/S0006-3495(68)86507-5 PMC1367399 5699795

[bb16] Haas, D. J. (1968*b*). *Acta Cryst.* B**24**, 604.

[bb17] Haas, D. J. (1969). *Monoclinic Lysozyme Crystals in Nonaqueous Liquids*. Abstract S-183. American Crystallographic Association.

[bb18] Haas, D. J. & Rossmann, M. G. (1968). *Reduced Radiation Damage in Lactic Dehydrogenase at −50°C*. Abstract N3, p. 60. American Crystallographic Association.

[bb19] Haas, D. J. & Rossmann, M. G. (1970). *Acta Cryst.* B**26**, 998–1004. 10.1107/s0567740870003485 5536135

[bb20] Harden, V. A. (2012). *AIDS at 30: A History*. Dulles: Potomac Books.

[bb21] Hope, H. (1988). *Acta Cryst.* B**44**, 22–26. 10.1107/s0108768187008632 3271102

[bb22] Hope, H. (1990). *Annu. Rev. Biophys. Biophys. Chem.* **19**, 107–126. 10.1146/annurev.bb.19.060190.000543 2194473

[bb24] King, M. V. (1958). *Nature*, **181**, 263–264.

[bb25] Low, B. W., Chen, C. C. H., Berger, J. E., Singman, L. & Pletcher, J. F. (1966). *Biochemistry*, **56**, 1746–1750. 10.1073/pnas.56.6.1746 PMC220166 16591415

[bb41] Navia, M. A., Fitzgerald, P. M. D., McKeever, B. M., Leu, C.-T., Heimbach, J. C., Herber, W. K., Sigal, I. S., Darke, P. L. & Springer, J. P. (1989). *Nature*, **337**, 615–620. 10.1038/337615a0 2645523

[bb27] Petsko, G. A. (1975). *J. Mol. Biol.* **96**, 381–392. 10.1016/0022-2836(75)90167-9 240944

[bb28] Pflugrath, J. W. (2015). *Acta Cryst.* F**71**, 622–642. 10.1107/S2053230X15008304 PMC4461322 26057787

[bb29] PhRMA (2018). *Falling Death Rates for HIV/AIDS Patients.* https://www.phrma.org/Graphic/Falling-Death-Rates-for-HIV-AIDS-Patients. Pharmaceutical Research and Manufacturers of America, Washington.

[bb30] Post, B., Schwartz, R. S. & Fankuchen, I. (1951). *Rev. Sci. Instrum.* **22**, 218–219.

[bb31] Quiocho, F. A. & Richards, F. M. (1964). *Biochemistry*, **52**, 833–839. 10.1073/pnas.52.3.833 PMC300354 14212562

[bb32] Rudman, R. (1976). *Low-Temperature X-ray Diffraction: Apparatus and Techniques*. New York: Plenum Press.

[bb40] Teng, T.-Y. (1990).* J. Appl. Cryst.* **23**, 387–391.

[bb33] Traub, W. & Hirshfeld, F. L. (1960). *Acta Cryst.* **13**, 753–760.

[bb34] Tulinsky, A. (1996). *Annu. Rep. Med. Chem.* **31**, 357–366.

[bb35] Wonacott, A., Mermall, H., Schevitz, R., Haas, D., McPherson, A. & Rossmann, M. G. (1968). *Fed. Proc.* **27**, 522.

[bb23] Wright, P. E. (2009). *J. Mol. Biol.* **392**, 1.

